# Novel Biomarker for the Diagnosis and Prognosis of Acute Alcoholic Hepatitis

**Published:** 2020-07-28

**Authors:** Vatsalya Vatsalya

**Affiliations:** 1 Department of Medicine, Division of Gastroenterology, Hepatology, and Nutrition, University of Louisville, Louisville KY USA; 2 Robley Rex VA Medical Center, Louisville KY USA; 3 University of Louisville Alcohol Research Center, Louisville KY USA

**Keywords:** Alcoholic hepatitis, Alcoholic liver disease, Alcohol use disorder, Non-invasive biomarker

## EDITORIAL

Alcohol use disorder (AUD) characterized by heavy and prolonged alcohol intake could cause various forms of alcoholic liver disease (ALD). The spectrum of alcoholic liver disease (ALD) consists of steatosis, steatohepatitis, cirrhosis and hepatocellular carcinoma depending on the duration and severity of alcohol intake [1,2]. Interestingly only one-third of heavy drinkers (typically a sub-group of AUD patients), who exhibit specific heavy drinking patterns [3,4] would develop any clinically relevant form of liver damage. However, only 10 to 15% of all drinkers develop severe forms of ALD. While numerous biomarkers have been identified with the diagnoses of early stage, and advanced form of ALD; no specific biomarker has described the staging, severity and prognosis clearly. Acute alcoholic hepatitis (AAH), an advanced form of ALD is a major cause of liver related morbidity and mortality. Established markers of liver damage (AST, ALT) are highly nonspecific and are affected by a wide array of pathologies. This creates a gap in the understanding of advanced forms of ALD, for example alcoholic hepatitis. New biomarkers are being tested to determine the degree of ongoing liver pathology and prognosis; and could be used in deciding the appropriate treatment. A non-invasive biomarker, extracellular cytokeratin 18 (K18) has recently been reported to have substantial association with the degree and severity of liver injury and liver cell death in ALD. Cytokeratin 18 (K18) is a death marker for epithelial cell, and their serum concentrations could be very high following hepatocyte death [5]. During cell death, loss of cell membrane integrity could be consequential in the release of intracellular proteins (including K18), into the extracellular compartment. K18 is a substrate cleaved by caspase-3, and the cleaved form of K18 is K18M30 that determines the degree of apoptosis. K18M65 is a biomarker for necrosis (both the caspase-cleaved and uncleaved forms). Both M65 and M30 can be detected in plasma using ELISA testing [6]. Bissonnette et al. [7] compared M65 and M30 levels to the histologically confirmed cases of alcoholic hepatitis with significantly positive results including a positive predictive value of 91% at a M65 cutoff of 2000 IU/L with a 81% accuracy in diagnosis. In a recent study, Keratin 18 appears to reflect the degree of hepatocyte death and delineate liver disease severity better than other traditional biomarkers, such as AST, ALT, and the AST:ALT ratio [8]. ALD is a leading cause of reversible morbidity and mortality. However, significant advancement is needed to characterize AAH presentation in context of the ongoing liver injury and liver cell death. Medical management of AAH will eventually involve the use of biomarkers (like K18) that could accurately reflect the clinical presentation for diagnosis, and prognosis of the disease severity.

## Abbreviations

AAH: Acute Alcoholic Hepatitis
ALD: Alcoholic Liver Disease
ALT: Alanine Aminotransferase
AUD: Alcohol Use Disorder
AST: Aspartate Aminotransferase
CK 18: Cytokeratin 18
